# Off-label use of thalidomide in children with Crohn's disease and inflammatory bowel diseases

**DOI:** 10.31744/einstein_journal/2025RW1270

**Published:** 2025-11-13

**Authors:** Laura Beatriz Fonseca, Paula Lana de Miranda Drummond, Cristine de Araújo Silva, Cristiane Aparecida Menezes de Pádua

**Affiliations:** 1 Universidade Federal de Minas Gerais Department of Social Pharmacy Belo Horizonte MG Brazil Department of Social Pharmacy, Universidade Federal de Minas Gerais, Belo Horizonte, MG, Brazil.; 2 Fundação Ezequiel Dias Belo Horizonte MG Brazil Fundação Ezequiel Dias, Belo Horizonte, MG, Brazil.

**Keywords:** Crohn's disease, Colitis, ulcerative, Inflammatory bowel diseases, Thalidomide, Efficacy, Safety, Off-label use, Child

## Abstract

Thalidomide exerts immunomodulatory properties that may be used to treat various conditions. Although its effectiveness in treating Crohn's disease in pediatric patients has been reported, its safety for off-label use is not well established. This study aimed to analyze the efficacy and safety of thalidomide in children and adolescents with inflammatory bowel disease, specifically Crohn's and ulcerative colitis. A narrative review of clinical trials and observational studies published between January 2001 and May 2023 that investigated thalidomide use in pediatric patients with Crohn's or ulcerative colitis was conducted. The results of clinical trials showed statistically significant remission rates and clinical improvement in patients. The most reported adverse event was peripheral neuropathy, resulting in discontinuation of medication in some cases. Other adverse effects, such as sedation, constipation, dizziness, and allergic reactions, were observed but were not serious. Thalidomide can be considered an alternative treatment option for pediatric patients with refractory inflammatory bowel disease. However, monitoring for peripheral neuropathy and potential dose adjustments are necessary. No new or unknown adverse reactions related to the medication were identified in this review.

## INTRODUCTION

Thalidomide is an anti-inflammatory, immunomodulatory, and anti-angiogenic drug first synthesized in 1954 and marketed in 1957 as a sedative and anti-emetic agent. After its release, fetal malformations, including phocomelia, dysmelia, amelia, bone hypoplasia, and congenital anomalies in organs, such as the ears and heart, were observed in infants born to women who had used the medicine during gestation. Consequently, the teratogenicity of thalidomide became apparent in the 1960s when several countries began its withdrawal from the market. Approximately 10,000 children worldwide were affected by complications of thalidomide use.^(1)^

Despite the tragedy associated with thalidomide, in 1969, Sheskin used thalidomide as a sedative in a patient with erythema nodosum leprosum (ENL) suffering from insomnia and observed an improvement in symptoms. In 1971, the World Health Organization coordinated a clinical study demonstrating significant improvements in several patients with ENL after thalidomide use. Therefore, thalidomide was approved for the treatment of ENL despite strict regulations and precautions.^(2-4)^

In Brazil, thalidomide is currently indicated for treating ENL, multiple myeloma, idiopathic aphthous ulcers in people living with HIV, graft-versus-host disease, lupus erythematosus, and myelodysplastic syndrome.^(5,6)^ As there is no information available on the pharmacokinetic parameters of the medicine for individuals under 18 years, the use of thalidomide is not permitted by regulatory agencies such as the National Health Surveillance Agency (ANVISA - *Agência Nacional de Vigilância Sanitária*) in Brazil, the United States Food and Drug Administration, and the European Medicines Agency.^(7)^

Thalidomide is a therapeutic option for the treatment of Crohn's disease (CD) in patients refractory to other treatments, owing to its immunomodulatory, anti-inflammatory, and anti-angiogenic properties. Thalidomide also has multiple mechanisms of action against inflammatory bowel disease (IBD). By inhibiting the production of tumor necrosis factor-alpha (TNF-α), which is a central pro-inflammatory cytokine in the pathogenesis of CD, thalidomide reduces the inflammatory response in the bowel. Additionally, thalidomide modulates the production of other pro-inflammatory cytokines, such as interleukin-1, interleukin-6, and interferon-gamma, as well as modulates the function of immune system cells, such as macrophages and T cells, reducing their exaggerated inflammatory response. Furthermore, thalidomide exhibits anti-angiogenic properties by inhibiting the formation of new blood vessels. Patients with CD have elevated levels of vascular endothelial growth factor and basic fibroblast growth factor, both of which are significantly correlated with disease activity and contribute to bowel inflammation and excessive scarring.^(8)^

Studies have demonstrated the potential efficacy of thalidomide in the treatment of CD and other IBD subtypes in adult and pediatric patients.^(9,10)^ However, there is limited data on the benefits and harm of thalidomide, and the treatment of children and adolescents is restricted to off-label use of the medicine.^(11)^

This study aimed to address an important gap in the understanding of pediatric IBD, particularly in the context of the limited data available on the efficacy and safety of thalidomide in pediatric patients with CD and ulcerative colitis (UC). This study offers a broader perspective on the adverse events (AEs) associated with thalidomide therapy. Consequently, this review adds to the medical literature on the comprehension and assessment of off-label thalidomide use in IBD and pediatric cohorts.

This study was a non-systematic review evaluating the efficacy, effectiveness, and safety of the off-label use of thalidomide in pediatric patients with IBD and included randomized clinical trials (RCTs), case reports, and case series on the off-label use of thalidomide in pediatric patients (0-18 years) with CD or UC. Therefore, a literature search was conducted using PubMed, SciELO, and Embase for studies published between 2001 and 2023.

This narrative review focused on studies addressing relevant aspects of the effectiveness and safety of therapeutic interventions in patients with CD, considering parameters such as remission rates, time to remission, disease maintenance, activity scores (such as CDAI), symptom improvement, and the occurrence of AEs. The extracted information emphasized key clinical outcomes, including symptom improvement, time to remission, and maintenance of therapeutic response. When applicable, data on AEs were compared with the safety information provided in the package insert of the Brazilian manufacturer.^(7)^

## AVAILABLE EVIDENCE ON THE OFF-LABEL USE OF THALIDOMIDE IN PEDIATRIC INFLAMMATORY BOWEL DISEASE

A total of 152 scientific articles were retrieved from the initial searches of PubMed (n=34), SciELO (n=0), and Embase (n=118). Of these, 29 articles were identified as duplicates, leaving 96 references for title and abstract screening. After reviewing titles and abstracts, 75 articles were excluded; 24 studies were selected for full-text reading and 14 were excluded. The primary reasons for exclusion were as follows: a) age >18 years, b) absence of reporting of AEs or documentation, and c) absence of efficacy/effectiveness results. The manual selection resulted in the inclusion of three references; thus, 10 articles (Kabuki et al.;^(12)^ Dipasquale et al.;^(13)^ Lazzerini et al.;^(14)^ Lazzerini et al.;^(15)^ Facchini et al.;^(16)^ Ahmed et al.;^(17)^ Lazzerini et al.;^(18)^ Felipez et al.;^(19)^ Wang et al.;^(20)^ Wang et al.;^(21)^ were selected for the review.

Among the 10 studies, 2 were RCTs,^(14,15)^ 6 were case series,^(12,17-20)^ and 2 were case reports,^(13,21)^ with a total sample size of 213 participants. The mean age when thalidomide treatment was initiated in pediatric patients ranged from 5 to 16 years. The articles obtained did not contain data on the minimum age for thalidomide treatment initiation, and the potential association between thalidomide use and developmental issues (considering its teratogenic effects during embryogenesis) was not mentioned (Table 1).

**Table 1:** Summary of studies included in the review of the use of thalidomide in pediatric inflammatory bowel disease

Study (year of publication)	Location of study	Study design	Sample (n)	Disease/condition	Mean age (years)*	Follow-up	Mean time to reach remission	Dose of thalidomide	Reduction or discontinuation of corticosteroids
Lazzerini et al.,^(14)^ 2013	Italy	Randomized clinical trial	56	CD	14.0 (2-18)	52 weeks	8 weeks	1.5-2.5mg/kg/day	All the children had ceased receiving steroids by week 16
Lazzerini et al.,^(15)^ 2015	Italy	Randomized clinical trial	26	UC	11.7 (2-18)	52 weeks	8 weeks	1.5-3.5mg/kg/day	NR
Facchini et al.,^(16)^ 2001	Italy	Case series	5	CD	17.0 (8-17)	24 months	3 months	1.5-2mg/kg/day	In two patients, steroids had been discontinued for more than 20 months
Ahmed et al.,^(17)^ 2003	United Kingdom	Case series	6	IBD	NR	3-6 months	3 months	50-200 mg/day	NR
Lazzerini et al.,^(18)^ 2007	Italy	Case series	28	IBD	16.4 (13-18)	36 weeks	8 weeks	1.5-2.5mg/kg/day	Sixteen of 20 (80%) patients suspended steroids
Felipez et al.,^(19)^ 2012	Italy	Case series	12	CD	10.0 (3-14)	4.6 years	2 months	50-150mg/day	Seven of 12 patients discontinued steroids (58.3%), 3 of 12 patients (25%) decreased their steroid dose by >50%, and 1 of 12 patients (8.3%) had no change in prednisone dose
Wang et al.,^(20)^ 2017	China	Case series	10	CD	7.2 (2-13)	22.2 months	9 months	1.2-2.5mg/kg/day	NR
Wang et al.,^(21)^ 2020	China	Case series	62	CD	9.4 (9-13)	30.5 months	6 months	1.5-2.5mg/kg/day	Most patients (95.4%, 21/22) discontinued steroids with a median time of 4.4 months

* Patient age at initiation of thalidomide treatment.

NR: not reported; CD: Crohn's disease; IBD: inflammatory bowel disease; UC: ulcerative colitis; AE: adverse events.

## EFFICACY AND EFFECTIVENESS

### Randomized clinical trials

Based on the RCTs obtained in the search, the use of thalidomide in pediatric patients with refractory CD and UC resulted in remission and maintenance of remission of the disease compared to placebo. Lazzerini et al.^(14)^ conducted a clinical trial involving 56 children with CD refractory to conventional treatment and assigned them to the thalidomide intervention or placebo groups. After 8 weeks, 13 of 28 children who received thalidomide (46.4%) achieved clinical remission compared to the placebo group (11.5%; n=26) (relative risk [RR]=4.0; 95% confidence interval [95%CI] 1.2-12.5; p=0.01; number needed to treat [NNT], 2.86 [95%CI=1.18-9.14]). Additionally, the mean CDAI, erythrocyte sedimentation rate, weight-for-age, and clinical global assessment scores were significantly higher in the thalidomide group than in the placebo group. Similarly, Lazzerini et al.^(15)^ conducted an RCT of 26 pediatric patients with refractory UC and assigned them to thalidomide (n=12) and placebo (n=11) intervention groups. In this study, all participants were refractory or intolerant to the recommended medications used in the treatment of UC, including corticosteroids and/or azathioprine. Approximately one-third of the participants had received infliximab, and another third had received cyclosporine. Within 8 weeks, thalidomide showed a conclusive benefit compared to placebo, with 10 of 12 children (83.3%) achieving clinical remission compared with 2 of 11 children (18.2%) in the placebo group (RR=4.5; 95%CI=1.2-16.4; p=0.005; NNT, RR=1.5; 95%CI=1.1-4.0). Clinical response was also significantly better at week 8 in the thalidomide group compared to the placebo group: 8/12 (66.6%) *versus* 2/11 (18.2%) (RR=3.7; 95%CI=1.0-13.7; p=0.03; NNT, RR=1.5; 95%CI=1.18-8.14). Furthermore, thalidomide was effective at low daily doses (1.2mg/kg/day), resulting in lower frequencies of dose-dependent AEs and allowing for long periods of remission before high cumulative doses were reached.

### Case series

Facchini et al.^(16)^ described a case series of thalidomide use in pediatric patients with CD who were intolerant or resistant to conventional treatments (immunobiologics, azathioprine, or methotrexate), or as a last resort before surgical intervention. Five patients received thalidomide at 1.5-2mg/kg/day, and administered at night. Clinical outcomes were assessed using the CDAI, HBI (Harvey-Bradshaw Index), and corticosteroid reduction rates. The results showed that in four of five patients, the mean CDAI and HBI scores decreased from 36.9 to 2.5 and from 8.5 to 0.75, respectively, after 3 months, suggesting a decrease in CD activity. Improvements in symptoms were also observed within 3-5 weeks. Ultimately, two patients discontinued corticosteroid use within 3 months, and two patients reduced their prednisolone dose starting from month 3 and discontinued it after 7 and 10 months. Endoscopic examination confirmed remission in all four patients, and none experienced relapse 19 months after the start of treatment. One patient eventually discontinued thalidomide treatment.

In the United Kingdom, Ahmed et al.^(17)^ reported their experience in treating two patients with CD using thalidomide and conducted a survey among major pediatric gastroenterology centers. The researchers distributed a questionnaire to members of the British Society of Pediatric Gastroenterology, Hepatology, and Nutrition and observed that immunomodulatory medication was used for the treatment of IBD in only six children. Indications included severe orofacial granulomatosis and/or refractory perianal CD unresponsive to conventional medical therapy. In the six cases, a single daily dose of thalidomide (50-200mg) was administered for 3-6 months, resulting in symptom improvement and a reduction in the need for steroid anti-inflammatory medications in three of six cases.

Lazzerini et al.^(18)^ described a sample of 28 children with moderate-to-severe IBD. Of these, 23 (82%) (18 participants with CD and 5 with UC) achieved disease remission within a median of 8 weeks (interquartile range [IQR]: 4-12 weeks) with thalidomide doses ranging from 1.5 to 2.5mg/kg daily. The median duration of remission was approximately 34.5 months (median, 29 months; IQR: 20-46). Another parameter observed in this study was that following remission, corticosteroid medications were either discontinued or their doses reduced. Among the 20 patients who received prednisone at the beginning of the study, treatment with anti-inflammatory medications was discontinued in 16 patients (80%); one patient was maintained on very low doses of prednisone (2.5 mg/day), and the median time required until corticosteroid discontinuation was 10 weeks (median, 12 weeks; IQR, 8-36 weeks). They concluded that thalidomide was effective at low daily doses (1.2mg/kg/day) and resulted in lower frequencies of dose-dependent AEs, allowing for long periods of remission before high cumulative doses were reached.

Felipez et al.^(19)^ also conducted a case series of patients with severe refractory CD who failed to respond to anti-TNF-α immunobiologics. Thalidomide treatment was prescribed to 12 patients with an average age of 10 years. Previous treatment included azathioprine/mercaptopurine (11/12 patients), methotrexate (7/12 patients), and anti-TNF-α immunobiologics (12/12 patients). The outcomes were assessed using the HBI score, reduction in corticosteroid dose, hospitalization, laboratory tests, and need for surgical intervention.

The mean treatment duration was 39.5 months, and the average HBI score was <5 in 10 patients (83.3%), indicating clinical remission. Five patients achieved fistula closure (5/7 patients; 71.4%), with the average prednisone dose decreasing from 13.9 to 2.3 mg daily, and seven patients discontinued corticosteroid use (7/12; 58.3%). Over a mean follow-up period of 4.6 years, researchers observed a decrease in the frequency of surgical procedures compared to the period prior to thalidomide treatment, wherein 6 patients (50%) underwent intestinal resections ranging from one to four procedures within a mean of 4 months (range: 3-96 months) from CD diagnosis to the initiation of thalidomide treatment. Meanwhile, two (2/12, 16.6%) patients required intestinal resection after thalidomide treatment. Five children (41.6%) had undergone incision and drainage prior to intervention treatment within a mean time from CD diagnosis to surgery of 12 months (range, 7-24 months); three of these patients required 2-3 procedures. Notably, none of the patients underwent any of these procedures while receiving thalidomide. The surgery rate was also calculated before and during thalidomide treatment, revealing a pre-thalidomide surgery rate of 26 surgeries (resections, incisions, drainage, and setons) in 826 patients (0.031), while it was 2 surgeries in 473.5 patient-months (0.004) during and after thalidomide treatment. The mean duration of thalidomide use was 39.5 months, the mean observation period before thalidomide use was 5.8 years, and the mean follow-up period after thalidomide use was 4.6 years (p=0.8).

### Case reports

Wang et al.^(20)^ reported that six patients (6/10; 60%) achieved clinical remission of CD with evidence of tuberculosis, and a clinical response was observed through a decrease in the CDAI score in three children (3/10; 30%) within 9-12 months after initiating thalidomide treatment. Similarly, Wang et al.^(21)^ reported the long-term experience of off-label thalidomide use in pediatric CD. They obtained clinical remission rates of 53.2% (33/62) at 6 months, 54.8% (34/62) at 12 months, and 33.9% (21/62) at the end of the follow-up period. The researchers also noted that most patients (95.4%, 21/22) discontinued corticosteroid therapy within a mean of 4.4 months.

Kabuki et al.^(12)^ described a case report of a patient diagnosed with CD at 6 months of age through endoscopic examination. As immunosuppressant medications were ineffective, thalidomide pharmacotherapy was administered as conventional treatment along with corticosteroids. Within 2 months of treatment, there was an improvement in diarrhea, abdominal pain, and sudden high fever, and the CDAI-based score significantly decreased from 45 to 15. However, thalidomide was discontinued because of the occurrence of AEs as described in section 3.2 of this study. Additionally, fistula closure resulting from CD was maintained after thalidomide withdrawal.

Dipasquale et al.^(13)^ reported a case of RU in a 2.1-year-old patient. The disease was refractory to corticosteroids, cyclosporine A, and infliximab, resulting in surgery (proctocolectomy with ileal J-pouch anal anastomosis and ileostomy) 9 months after diagnosis. After ileostomy reversal, the patient experienced abdominal pain, frequent watery bowel movements throughout the day, and hematochezia. Various pharmacotherapies containing antimicrobials (ciprofloxacin and metronidazole) resulted in initial improvement but were ineffective thereafter. After 3 months, azathioprine 2mg/kg/day was initiated, leading to partial clinical benefits. During a repeat endoscopy examination at 5 years, multiple aphthous lesions and linear ulcers were observed in the intestine. Histological examination revealed non-caseating granulomas with consistent active chronic colitis, indicative of CD. The patient promptly started therapy with prednisone 1mg/kg/day and metronidazole while continuing azathioprine. His symptoms significantly improved but recurred after 3 months. Based on the medical history, thalidomide 2mg/kg/day was initiated, resulting in symptom resolution and visual improvement on endoscopic examination. After 6 months of thalidomide treatment, the patient remained in good clinical condition, with 5-6 bowel movements per day and no hematochezia or abdominal pain. The patient underwent neurological, psychological, and electromyographic examinations to rule out peripheral neuropathy; the results were within normal limits.

## SAFETY

Table 2 presents the frequency of AEs reported in the included studies. There is a higher number of reports involving the development of peripheral neuropathy, as confirmed by alterations in electromyography (EMG) exams. Peripheral neuropathy is considered a serious AE, and in some cases, a reason for treatment discontinuation and loss to follow-up. Additionally, constipation, drowsiness, allergic reactions, and dizziness were frequently observed.

**Table 2:** Frequency and distribution of adverse events associated with thalidomide use in pediatric patients with inflammatory bowel disease

Adverse events	Facchini et al.,^(16)^ 2001	Ahmed et al.,^(17)^ 2003	Lazzerini et al.,^(18)^ 2007	Felipez et al.,^(19)^ 2012	Lazzerini et al.,^(14)^ 2013	Lazzerini et al.,^(15)^ 2015	Wang et al.,^(20)^ 2017	Wang et al.,^(21)^ 2020
	% (n/total adverse events)							
Abnormal electromyography	-	-	25 (7/28)	-	48 (16/33)	26 (6/23)	-	27 (17/62)
Acne	-	-	-	-	-	4 (1/23)	-	-
Agitation	-	-	4 (1/28)	-	-	-	-	-
Alopecia	-	-	-	-	3 (1/33)	30 (7/23)	-	-
Amenorrhea	-	-	-	-	3 (1/33)	13 (2/23)	-	-
Amnesia	-	-	-	-	3 (1/33)	-	-	-
Anorexia	-	-	-	-	3 (1/33)	4 (1/23)	-	2 (1/62)
Anxiety	-	-	-	-	-	4 (1/23)	-	-
Asthenia	-	-	-	-	3 (1/33)	9 (2/23)	-	-
Body aches	-	-	-	-	-	-	10 (1/10)	8 (5/62)
Bradycardia	-	-	-	-	9 (3/33)	4 (1/23)	-	-
Cataracts	-	-	-	-	-	-	-	3 (2/62)
Conjunctivitis	-	-	-	8 (1/12)	-	4 (1/23)	-	-
Constipation	-	-	-	-	12 (4/33)	22 (5/23)	-	-
Depression	-	-	-	-	3 (1/33)	-	-	-
Dermatitis	-	-	11 (3/28)	-	30 (10/33)	-	-	5 (3/62)
Difficulty concentrating	-	-	-	-	3 (1/33)	4 (1/23)	-	-
Dizziness	-	-	4 (1/28)	8 (1/12)	-	4 (1/23)	-	5 (3/62)
Drowsiness	-	-	14 (4/28)	-	9 (3/33)	17 (4/33)	10 (1/10)	-
Dry eyes	-	-	-	-	-	-	20 (2/10)	-
Dysmenorrhea	-	-	-	-	3 (1/33)	-	-	-
Elevated liver enzymes	-	-	-	-	-	-	-	6 (4/62)
Fatigue	-	-	-	-	-	-	-	3 (2/62)
Gynecomastia	-	-	4 (1/28)	-	-	-	-	-
Hallucinations	-	-	4 (1/28)	-	-	-	-	-
Headache	-	-	-	-	9 (3/33)	17 (4/33)	-	-
Hemianopsia	-	-	-	-	3 (1/33)	-	-	-
Leukopenia	-	-	-	-	9 (3/33)	4 (1/23)	-	2 (1/62)
Lipothymia	-	-	-	-	-	4 (1/23)	-	-
Nausea	-	-	-	-	-	4 (1/23)	-	-
Optic neuritis	-	-	-	-	-	4 (1/23)	-	-
Peripheral neuropathy	20 (1/5)	66 (4/6)	25 (7/28)	41 (5/12)	21 (7/33)	26 (6/23)	-	-
Photophobia	-	-	-	-	-	4 (1/23)	-	-
Scalp psoriasis	-	-	-	-	3 (1/33)	-	-	-
Sedation	-	-	14 (4/28)	-	-	-	-	-
Seizure	-	-	-	-	3 (1/33)	-	-	-
Tremors	-	-	-	-	-	-	-	5 (3/62)
Tricuspid regurgitation	-	-	-	-	-	-	-	2 (1/62)
Urticaria	-	-	-	-	-	4 (1/23)	-	-
Worsening of IBD	-	-	-	17 (2/12)	-	-	-	-

IBD: inflammatory bowel disease.

In an RCT with children and adolescents refractory to CD, Lazzerini et al.^(14)^ observed AEs in 14 of 49 (28.5%) and 1 of 26 (3.8%) patients in the treatment and placebo groups, respectively (p=0.009). During a follow-up period of 4,025 patient-weeks, there were nine severe AEs that required treatment discontinuation, with a cumulative incidence of 2.1 per 1,000 patient-weeks (95%CI=1.1-4.1). Peripheral neuropathy was the most frequently reported AE, with clinical neuropathy being associated with a cumulative dose of 380mg/kg (10 months of thalidomide therapy). There were also indirect indications of neuropathy, including mild alterations in EMG exams along with mild clinical manifestations, with a cumulative incidence of 1.2 per 1,000 patient-weeks (95%CI=0.4-2.7), and isolated alterations in EMG exams were considered relatively common (2.7 per 1,000 patient-weeks; 95%CI=1.4-4.7). With the reduction in thalidomide doses, improvement or stabilization of clinical manifestations and EMG changes related to peripheral neuropathy were observed in approximately half of the children who presented with the condition. Other severe AEs included amenorrhea, bradycardia, and a case of an acute neurological event, which was interpreted as possible migraine or transient ischemic attack. Non-severe AEs had a total cumulative incidence of 12.2 cases per 1,000 patient-weeks (95%CI=9-15).

During 8 weeks of follow-up, Lazzerini et al.^(15)^ reported that 13 of 23 (56%) patients with refractory UC on thalidomide treatment and 2 of 11 (18%) children in the placebo group experienced AEs. However, none of the cases required treatment discontinuation. Among the reported AEs, dermatitis was most frequently observed (21.7%), followed by headache (13.0%) and isolated neurological symptoms without EMG changes (8.7%). Notably, all AEs have been described in the literature and in Brazilian, European, and American manufacturers’ leaflets. However, there was an increase in the frequency of long-term thalidomide-related AEs (mean of 109.9±108.7 weeks). Therefore, eight AEs were recorded as causes of treatment discontinuation, with a cumulative incidence of 3.1 per 1000 patient-weeks (95%CI=1.5-6.0). In contrast, peripheral neuropathy was the most frequently observed AE, related to a cumulative dose of 332 mg/kg (36 weeks). Neuropathy was reported in six patients (6/23; 2.3 per 1,000 patient-weeks), dermatitis was the most frequently reported AE (7/23; 2.8 per 1,000 patient-weeks), and constipation was the third most frequently registered AE (5/23; 2.0 per 1,000 patient-weeks).

Lazzerini et al.^(18)^ conducted a case series of 28 pediatric patients with refractory IBD. Overall, seven (25%) patients discontinued thalidomide treatment because of peripheral neuropathy. There were no cases of neuropathy in patients who received a cumulative thalidomide dose of <28g (IQR=42-70g). All patients with neuropathy showed complete recovery after thalidomide discontinuation or resolution of symptoms after dose reduction. However, there were other the reasons for thalidomide discontinuation. One patient experienced dizziness and drowsiness and did not tolerate the medication well, and another experienced agitation with hallucinations and concomitant neuropathy. Other AEs that did not result in thalidomide discontinuation included mild sedation (4/28, 14.28%), dizziness (1/28, 3.57%), dermatitis/pruritus (3/28, 10.71%), and gynecomastia (1/28, 3.57%). Among these, gynecomastia alone was not described as an adverse reaction in the approved package inserts of thalidomide manufacturers.

Facchini et al.^(16)^ described five patients using thalidomide for CD that was refractory to conventional immunobiologics, azathioprine, or methotrexate. One patient discontinued treatment after 6 days because of distal paresthesia in both feet; results for nerve conduction alteration were negative, indicating peripheral neuropathy. Similarly, Ahmed et al.^(17)^ described a case series on thalidomide use in children with CD throughout the UK, with the aim of reporting cases of peripheral neuropathy. Despite the reduction in symptoms and decreased need for corticosteroids, four of six patients developed peripheral neuropathy (considered a serious AE), leading to treatment discontinuation; this was a major factor limiting thalidomide use in the UK. Therefore, researchers suggest that thalidomide be used with caution in carefully selected patients with severe CD refractory to conventional treatments. Furthermore, they emphasized the need for nerve conduction speed monitoring tests every 3 months during treatment, clinical monitoring, and providing guidance to the parents or guardians of the patient on the management of neuropathy.

Felipez et al.^(19)^ obtained nerve conduction test results from five patients (5/12). Of these, the results of four patients were consistent with mild sensory neuropathy, while that of one patient showed a moderate presentation. Among the cases of mild neuropathy, resolution was documented by EMG after reducing the thalidomide dose. Additionally, resolution of symptoms and laboratory signs of peripheral neuropathy occurred within 2-3 months after the discontinuation of medication or dosage reduction. In the children who developed neuropathy, the average cumulative dose of thalidomide was 54.4g (16.5-102.8g) vs. 49.0g (1.5-166.0g) in those with symptoms or clinical findings indicative of peripheral neuropathy (p=0.85). The main reasons for treatment discontinuation were peripheral neuropathy (42%, 5/12), worsening CD (17%, 2/12), dizziness (8%, 1/12), and eye allergies (8%, 1/12). Furthermore, 3 of 12 (25%) patients continued thalidomide treatment for a mean of 72 months (range, 48-96 months).

According to Wang et al.,^(20)^ the safety of the medication was well established during a median follow-up period of 22.2 months, with a low incidence of AEs in children with CD and concurrent tuberculosis infection. The researchers reported only one case of drowsiness, one case of dry eyes, and a case of a patient experiencing dry eyes and knee pain (recovering after discontinuation of thalidomide and subsequent administration without AEs). No symptoms or signs of sensory impairment were observed during the follow-up period.

In a retrospective study based on the observation of long-term clinical outcomes of thalidomide use in pediatric patients with CD, Wang et al.^(21)^ achieved more conclusive results regarding AEs and, consequently, the safety of off-label treatment. The researchers recorded the occurrence of at least one AE in 45.2% (28/62) of the participants that manifested as either clinical symptoms or abnormalities on EMG. Additionally, 22 patients discontinued thalidomide because of AEs, with EMG abnormalities observed in 16 patients. Peripheral neuropathy was the most frequent AE, and all patients with neuropathy recovered after thalidomide discontinuation. The mean cumulative thalidomide dose was 32.3g in patients with abnormal EMG results vs. 34.4g in 10 patients with no EMG changes, suggesting that neuropathy occurred independently of the cumulative thalidomide dose (95%CI=32.3-30.8 *versus* 34.4-21.2; p=0.60). Nevertheless, there were other AEs, including back or limb pain (8%, 5/62), tremors (3/62, 4.8%), cataracts (2/62, 3.2%), tricuspid regurgitation (1/62, 1.6%), and leukopenia (1/62; 1.6%) in a patient with monocytic leukemia. When comparing the occurrence of AEs overall, the cumulative thalidomide dose was significantly different between the AE and non-AE groups (95%CI=37.8-30.1 *versus* 21.0±19.1; p=0.01).

In case reports, the safety profile of thalidomide was similar to that reported in RCTs and case series. Kabuki et al.^(12)^ observed edema, skin rash, and peripheral neuropathy after 4.5 months of thalidomide therapy in a child with CD, leading to treatment discontinuation. In contrast, Dipasquale et al.^(13)^ reported that their patient underwent EMG examinations to assess possible abnormalities related to peripheral neuropathy; however, the findings were normal. Therefore, no adverse reactions were described in this report.

Other adverse reactions described as very common (>10%) in the national manufacturer's leaflets for thalidomide include constipation, drowsiness, and hematological effects. From the two controlled clinical trials and two of six case series,^(12,16-20)^ frequencies within the expected range were observed for constipation and drowsiness. However, Wang et al.^(20)^ reported a frequency of 10% for drowsiness, classifying the reaction as common (>1% and ≤10%). The hematological effects in the reference studies were leukopenia^(10,11,19)^ and elevated liver enzyme levels. However, it was not possible to establish a frequency of adverse reactions exceeding 10% in the affected children.

The relationship between the cumulative dose of thalidomide and peripheral neuropathy varies among studies. In two clinical trials, medication-related AEs developed with a minimum cumulative dose of 332mg/kg (36 weeks of thalidomide therapy); however, most patients received very high cumulative doses without developing neuropathy.^(14)^ Furthermore, in a study conducted by the same group of researchers,^(15)^ peripheral neuropathy, observed through clinical examination, occurred with a minimum dose of 380mg/kg (10 months of therapy for pediatric patients with UC). Most patients receive very high cumulative doses of thalidomide without developing clinical neuropathy.

According to some authors, the hypothesis is that AEs are not clearly related to the dose of thalidomide. However, it appears to be cumulative dose-dependent, which allows for the use of low daily doses, resulting in long-term remission being achieved before high exaggerated doses are obtained.^(18)^

## LONG-TERM CONSIDERATIONS ABOUT THALIDOMIDE OFF-LABEL USE IN PEDIATRIC INFLAMMATORY BOWEL DISEASE

Despite not being approved for the treatment of IBD both in Brazil and internationally, this review demonstrates that there are publications justifying the off-label use of thalidomide in children with CD and UC. Controlled clinical trials involving pediatric patients with IBD have shown thalidomide-induced remission in 46.4% of patients with CD and 83.3% of patients with UC within 8 weeks, compared to 11.5% and 18.8%, respectively, in the placebo groups.^(12,13)^ However, despite evidence of the efficacy and effectiveness of thalidomide for refractory pediatric CD, there are restrictions on its use because of its numerous adverse reactions and teratogenicity.^(11)^ This review confirms the findings of a systematic review by Qiu et al.^(10)^ involving pediatric populations living with IBD and aligns with the established safety profile of thalidomide in adults. Thalidomide has shown efficacy in the long-term treatment of CD and other subtypes of IBD such as UC.^(10)^

Evidence on this topic is scarce; therefore, the results should be interpreted with caution. Inflammatory bowel disease is a multifactorial condition, and the disease course can vary with acute flares, episodes of remission, and symptom exacerbation, making spontaneous remission unlikely. Furthermore, episodes of disease relapse after thalidomide discontinuation, as observed by Lazzerini et al.,^(18)^ highlight its potential benefit in maintaining remission. In a study conducted by Wang et al.,^(21)^ a subset of patients (19.4%, 12/62) who received long-term thalidomide treatment experienced relapse after medication discontinuation at a mean of 23 months.

The reduction in the use of steroidal anti-inflammatory medications and the maintenance of long-term remission are noteworthy points as they are related to developmental disorders in pediatric patients. A decrease in the use of this medication, especially among patients with refractory IBD, has also been reported after thalidomide treatment.^(20)^ Furthermore, Felipez et al.^(19)^ noted significant improvements in all parameters established to measure the effects of thalidomide in pediatric patients with severe and refractory CD, along with long-term improvement and maintenance of remission (4.6 years).

A more detailed understanding of AEs can be obtained from clinical trials, series, and case reports, which are described along with quantitative frequency data. Therefore, adverse reactions to thalidomide described in the medication package inserts are in line with the results of clinical studies and available evidence, as the information in the package inserts is based on previous studies. The safety profile was similar to the records published in the articles and, consequently, to the medication package inserts. The most severe and frequent adverse reaction in pediatric patients is peripheral neuropathy, which is diagnosed based on abnormalities in EMG, symmetric sensory movement disorders, numbness in the upper and lower limbs, tingling, and muscle weakness.

In a systematic review involving 192 pediatric and adult patients with IBD^(9)^ treated with thalidomide, the most commonly reported AEs were sedation (32.3%), peripheral neuropathy (19.8%), and dermatitis (12%). Peripheral neuropathy was the most common reason for thalidomide discontinuation (14.6%). Overall, 29% of the patients discontinued thalidomide therapy at some point because of AEs, demonstrating the similarity between the findings of the present study and the available literature. Furthermore, peripheral neuropathy is described as a very common adverse reaction (>10%) in the national manufacturer's package insert. Frequencies of >10% in patients with neuropathy and abnormalities in EMG were observed in five articles,^(14,15,18,20,21)^ and frequencies >10% were also observed in six studies.^(10-15)^ Neuropathy was reversible in 100% of the pediatric patients included in the RCTs, series, and case reports in this narrative review. No new, unknown, or unreported adverse reactions related to the medication were observed in the literature or in the manufacturer's instructions. Adverse events, such as gynecomastia and hemianopsia, were attributed to a causal relationship with treatment by researchers or healthcare professionals who monitored the patients.

Although the relationship with the thalidomide dose is not clearly understood, it appears to be cumulative dose-dependent, allowing for the use of low daily doses and resulting in long-term remission before reaching high doses.^(10)^ Moreover, all observed AEs have already been described in the literature and, consequently, in the leaflets of national and international manufacturers. Another study showed that patients with polyneuropathies received cumulative thalidomide doses ranging from 1.4 to 207.7g, and all patients who received doses >60g developed neuropathy.^(22)^ The recommended starting oral dose of thalidomide, according to the drug labels, is 50mg/day for adults, with the possibility of gradually increasing the dose based on disease response and patient tolerance. The European Society for Pediatric Gastroenterology, Hepatology, and Nutrition and the European Crohn's and Colitis Organization suggest that a dose of 2 mg/kg/body weight may be appropriate for adolescents with CD, and lower doses may be considered for young children. However, the studies included in this review did not address the well-established daily dose of thalidomide in the pediatric population.

## LIMITATIONS, STRENGTHS, AND PERSPECTIVES

This review had some limitations owing to the low quality of evidence obtained from the included studies. First, only two small-scale RCTs were included; other studies were retrospective studies and case reports. Regarding case series and case reports, there was no control group for intervention comparison, making data collection difficult, and follow-up information may be compromised.

Nevertheless, case series and case reports can provide information on treatment success and safety profiles. The possibility of introducing immunobiologics has not yet been proposed in any of the studies. Third, owing to the difficulty of obtaining a homogeneous population (as it involves pediatric patients), the studies had small sample sizes for both the intervention and comparison groups. Furthermore, the studies included in this review originated in Europe and Asia, reducing the chances of observing the characteristics of the disease and treatment in different patient profiles.

Additionally, AE classification was heterogenous, as each research group had different methods. Adverse events can have multiple underlying factors (not always determined), and there are different ways of detecting an AE. Thus, varied frequencies are generated.

One of the strengths of this review is its ability to summarize current evidence regarding the use of thalidomide in rare diseases affecting the pediatric population, a group often underrepresented in randomized clinical trials. Thus, it provides valuable insights into an effective alternative therapeutic option for pediatric patients who should be carefully monitored to avoid the harmful effects of thalidomide treatment.

More robust RCTs involving larger populations should be conducted to add to the evidence on the efficacy and safety of thalidomide in pediatric patients refractory to treatment, those with different degrees of CD involvement, and those with UC. Long-term studies would also provide more information regarding the relationship between neuropathy and thalidomide dose.

Despite its therapeutic potential, the use of thalidomide requires rigorous monitoring because of the associated risks and teratogenicity. Therefore, thalidomide use should be guided in adolescent patients, as there is a risk of pregnancy. Additionally, peripheral neuropathy, although considered a serious AE, is reversible in some cases and requires proper guidance and timely dose adjustments. Patients using thalidomide should be frequently monitored for symptoms of neuropathy such as tingling, paresthesia, numbness, and weakness. Neurological examinations are also important in the event of condition development, and regular neurological examinations and assessment of vibration sensitivity every 6 months are indicated for individuals.^(11)^

## FINAL COMMENTS

Thalidomide appears to be an alternative option for pediatric patients with refractory IBD, as it induces disease remission. Peripheral neuropathy was the most frequently observed AE, and its frequency was similar to that in the available data on adult patients. Neuropathy was reversible in all affected patients but required monitoring and, in some cases, dose adjustments. Furthermore, no additional AEs beyond those previously described in the literature were identified. However, evidence regarding the use of thalidomide in pediatric populations with IBD is limited owing to several factors, including the scarcity of RCTs with large sample sizes, low prevalence of the disease in pediatric populations, and limited availability of well-structured and robust observational studies.
